# Olfactory Neuroblastomas: What Actually Happens in the Long-Term?

**DOI:** 10.3390/jcm11092288

**Published:** 2022-04-20

**Authors:** Konstantinos Mantsopoulos, Michael Koch, Heinrich Iro, Jannis Constantinidis

**Affiliations:** 1 Department of Otorhinolaryngology, Head and Neck Surgery, University of Erlangen–Nürnberg, 91054 Erlangen, Germany; michael.koch@uk-erlangen.de (M.K.); heinrich.iro@uk-erlangen.de (H.I.); 2 1st Department of Otolaryngology, Head & Neck Surgery, Aristotle University of Thessaloniki, 54636 Thessaloniki, Greece; janconst@otenet.gr

**Keywords:** olfactory neuroblastoma, esthesioneuroblastoma, Kadish, Hyams, recurrence, survival, endoscopic surgery

## Abstract

Objective: The aim of this study was to investigate the long-term oncologic outcome and review the state of the art in the management of olfactory neuroblastomas. Material and Methods: The records of all patients treated for olfactory neuroblastomas in two academic departments between 1975 and 2012 were evaluated retrospectively. Data on epidemiological parameters were collected (age, gender), along with staging (Kadish, Morita), histologic grading (Hyams), time and form of treatment, locoregional control, and disease-specific and overall survival. Patients with other malignant diseases, distant metastases of olfactory neuroblastomas at the time of initial diagnosis, a follow-up time of less than 5 years, or insufficient clinical-pathological data were excluded from further analysis. Results: In total, 53 cases made up our final study sample (26 men, 27 women; male–female ratio 0.96:1). Their mean age was 48.6 years (range: 10–84 years). The mean follow-up time was 137.5 months (4–336 months, SD: 85.0). A total of 5 out of 53 study cases (9.4%) showed metastatic involvement of the neck at the time of initial presentation. Local recurrence was detected in 8/53 (15.1%) and regional recurrence in 7/53 of our study cases (13.2%). Three patients (42.8%) from the group of cases with surgery as the sole form of management (7/53, 13.2%) died due to the disease. The cumulative disease-specific survival and overall survivalfor the whole group of patients were 88.6% and 63.6%, respectively. The cumulative disease-specific survival stratified by Kadish A/B vs. Kadish C/D as well as Hyams I/II vs. Hyams III/IV showed superior results for limited tumors, albeit without significance, and low-grade tumors (highly significant difference). Conclusion: Craniofacial or sometimes solely endoscopically controlled resection can warrant resection of the olfactory neuroblastoma with wide margins. However, locoregional failures and distant metastases can occur after a long period of time. The non-negligible incidence of regional recurrences, partly in unusual localizations, leads us to consider the need to identify the “recurrence-friendly” cases and to perform individualized elective irradiation of the neck in cases with high-risk features.

## 1. Introduction

The neuroectodermal malignancy of olfactory neuroblastoma (ON), which was discovered less than 100 years ago and initially named “*esthesioneuroepitheliome olfactif*” [1], has an incidence of about 0.4 cases per million and accounts for 3–6% of all sinonasal malignancies [2]. The vague symptomatic and demanding anatomic localization (“rhino-neurosurgical border”), the extremely variable and hardly predictable biologic behavior [3,4,5] (cases with slow evolution and late recurrences as well as aggressive and metastatic forms with fulminant behavior already at onset [6,7]) in combination with the extremely low prevalence constitute the challenging profile of this lesion. In our view, almost no other malignant entity in the head and neck region is complicated by such a large number of open, controversial clinical and surgical issues: the various staging systems [4,8,9,10,11,12,13,14] (as well as the absence of an official AJCC (The American Joint Committee on Cancer)/UICCC (Union internationale contre le cancer) staging system [11]); the debatable prognostic role [13,15,16,17]; the subjectivity as well as the sampling dependence [18] of histopathology-based Hyams grading; the possibility of reducing therapeutic invasiveness (single-modality surgical treatment) in carefully selected “low-risk” cases [19]; the role of local irradiation as well as elective nodal irradiation of a cN0 neck in “low-risk” lesions [7,20,21], e.g., in teenagers and young adults [6]; and the ideal imaging modality for the follow-up [22] as well as the long-term course of the disease [12,23] dominate the relevant literature. In the last three decades, the establishment of endoscopically controlled approaches (as the first or sole surgical step in tumors confined to the nasal cavity) as well as new irradiation modalities (e.g., intensity-modulated radiation therapy) opened new horizons in the “quality-of-life”-oriented but still oncologically sufficient management of these tumors [24,25].

The aim of this study was to present the experience of two academic centers in the long-term outcome of patients with ON over a period of 42 years (1975–2017) with a minimum follow-up of 5 years as well as review the state of the art in the relevant literature regarding the aforementioned controversial clinical and therapeutic issues of this demanding entity. The motivation behind this study lay in the need to optimize our patient counseling by enriching it with long-term feedback and perhaps by thoroughly reconsidering our management philosophy.

## 2. Materials and Methods

This study was performed at two academic tertiary referral centers (Department of Otorhinolaryngology, Head and Neck Surgery, University of Erlangen–Nuremberg, Erlangen, Germany and Department of Otorhinolaryngology, Head and Neck Surgery, University of Thessaloniki, Thessaloniki, Greece). The records of all patients treated curatively for ON before 2012 were studied retrospectively. Patients with other malignant diseases, distant metastases of ON at the time of initial diagnosis, a follow-up time of less than 5 years, or insufficient clinical-pathological data were excluded from further analysis. Data was collected on epidemiological parameters (age, gender), staging (Kadish [8], modified Kadish–Morita [26]), histologic profile (Hyams [13]), time and form of management, locoregional control, and disease-specific and overall survival. The specimens of all cases managed before introduction of Hyams’ grading in 1988 [13] were evaluated retrospectively for histopathologic grading from an experienced head and neck pathologist in our department. Staging was performed using information from patients’ surgical archives or imaging data (CT and/or MRI). The five-year overall survival estimate (OS) was defined as the percentage of patients who were still alive within 5 years divided by the total number of patients. The five-year disease-specific survival rate estimate (DSS) was defined using the time from the date of diagnosis to death from the cancer or from complications of treatment. Regional recurrence was defined as histologically confirmed ON in the neck after completion of initial treatment. DSS and OS were calculated using the Kaplan–Meier method. Univariate comparisons between subgroups were performed using the Log-Rank test. The association of 5-year-DSS with Kadish–Morita staging, Hyams grading, and initial N status was examined by means of multivariate linear regression analysis. A *p*-value < 0.05 indicated statistical significance. SPSS for Windows v. 25.0 (SPSS, Inc., Chicago, IL, USA) was used for statistical analysis. Approval was obtained from the institutional review boards of both hospitals.

## 3. Results

**Figure 1:**
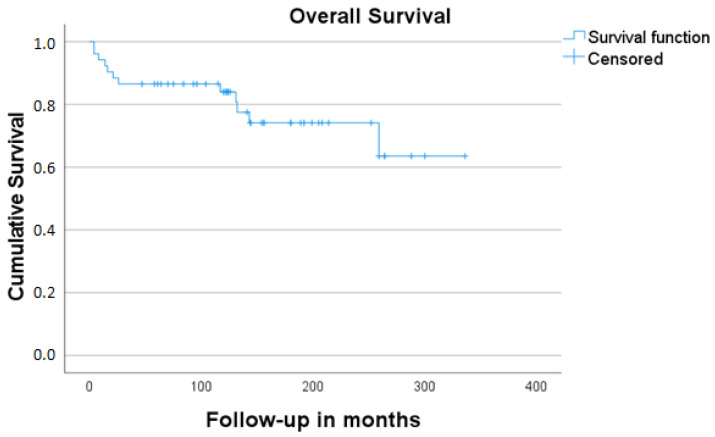
Overall survival estimates for all patients included in the study.

**Figure 2:**
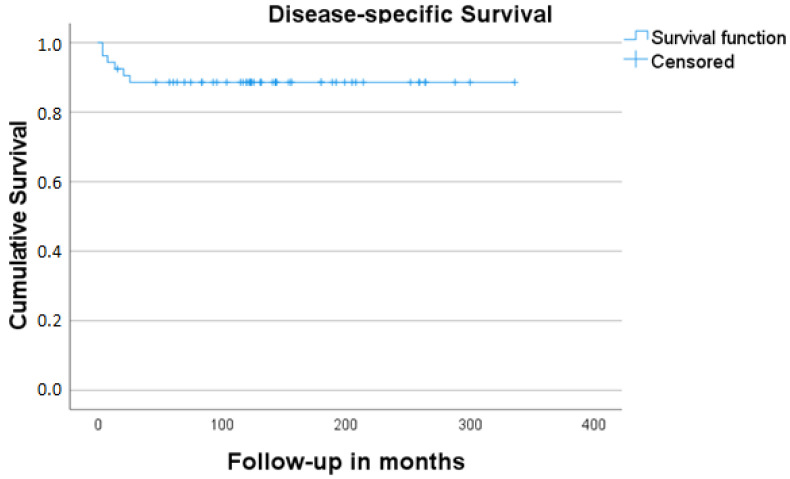
Disease-free survival estimates for all patients included in the study.

**Figure 3:**
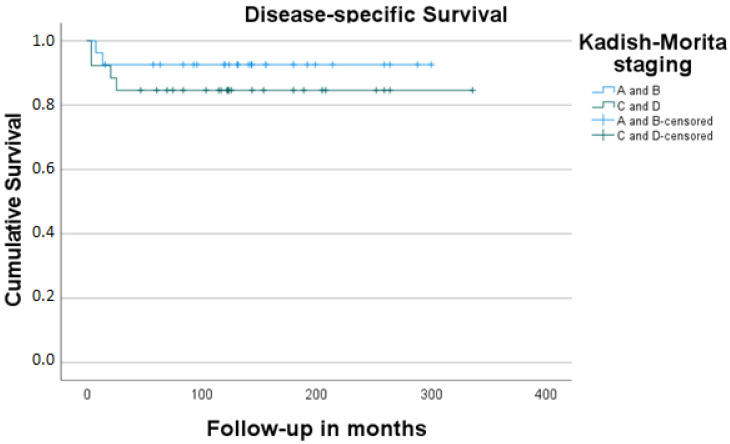
Estimated disease-free (D) survival stratified by Kadish–Morita stage.

**Figure 4:**
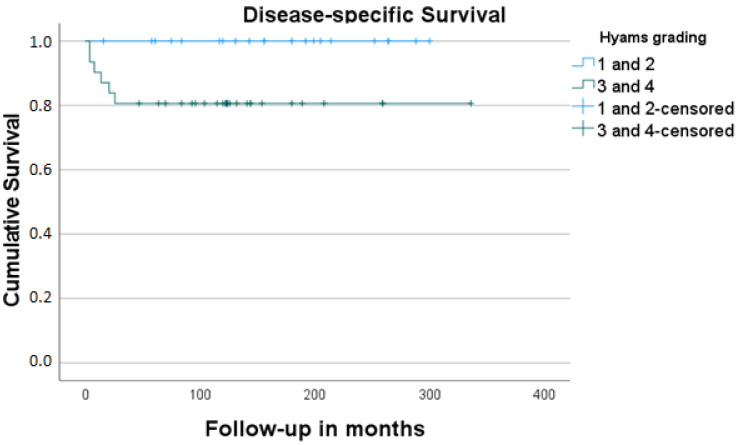
Estimated disease-free survival stratified by histologic grading (Hyams).

In total, 53 cases made up our final study sample (26 men, 27 women; male–female ratio 0.96:1). Their mean age was 48.6 years (range: 10–84 years). The mean follow-up time was 137.5 months (4–336 months, SD: 85.0). Detailed information on patients’ demographics, tumor characteristics, treatment form, and oncologic outcome is given in Table 1 and Table 2. A total of 5 out of 53 study cases (9.4%) showed metastatic involvement of the neck at the time of initial presentation. Local recurrence was detected in 8/53 (15.1%) and regional recurrence in 7/53 of our study cases (13.2%). Three patients (42.8%) from the group of cases with surgery as the sole form of management (7/53, 13.2%) died of the disease. The cumulative 5-year-DSS and OS for the whole group of patients were 88.6% and 63.6%, respectively Figure 1 and Figure 2. The cumulative DSS stratified by Kadish A/B vs. Kadish C/D as well as Hyams I/II vs. Hyams III/IV showed superior results for limited tumors, albeit without significance, and low-grade tumors (highly significant difference, Table 3, Figure 3 and Figure 4). Multivariate linear regression analysis showed that among the examined factors (Kadish–Morita staging, Hyams grading, initial N status), only Hyams grading was an independent prognostic for survival (*p* = 0.05). The 5-year-DSS was significantly higher in the group of patients treated in the 2001–2017 period compared to the patients treated in the 1975–2000 period (100% vs. 72.2%, *p* = 0.002).

## 4. Discussion

**Figure 5:**
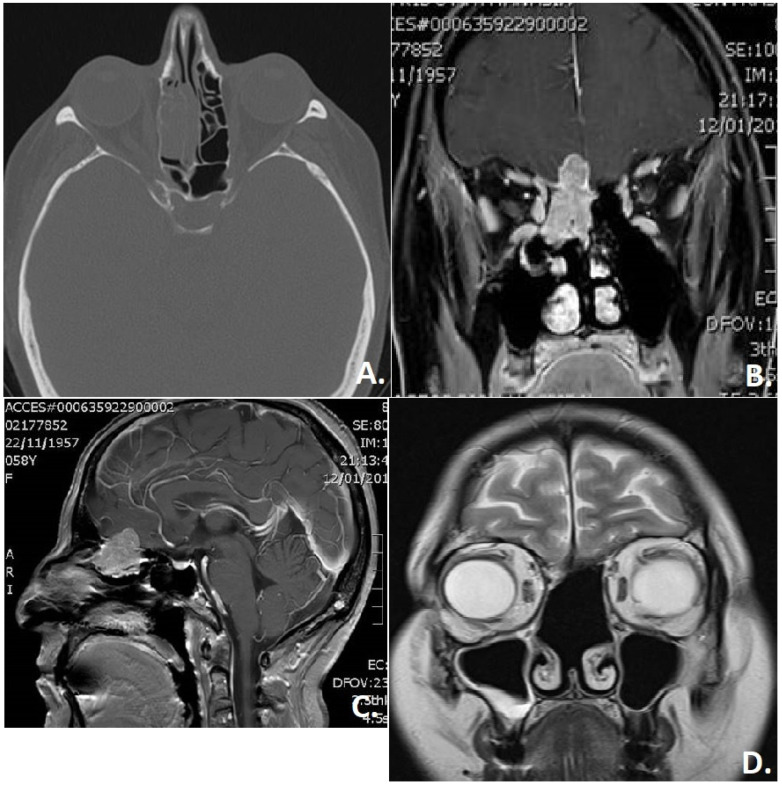
Imaging of a patient with an olfactory neuroblastoma. (**A**) Computed tomography (axial section) shows involvement of the ethmoid cells on the right side. (**B**) Magnetic resonance imaging (coronal section) and (**C**) sagittal section shows a marked “nodular” intracranial extension of the tumor. (**D**) Follow-up: magnetic resonance imaging (coronal section) without sign of local recurrence on follow-up.

**Figure 6:**
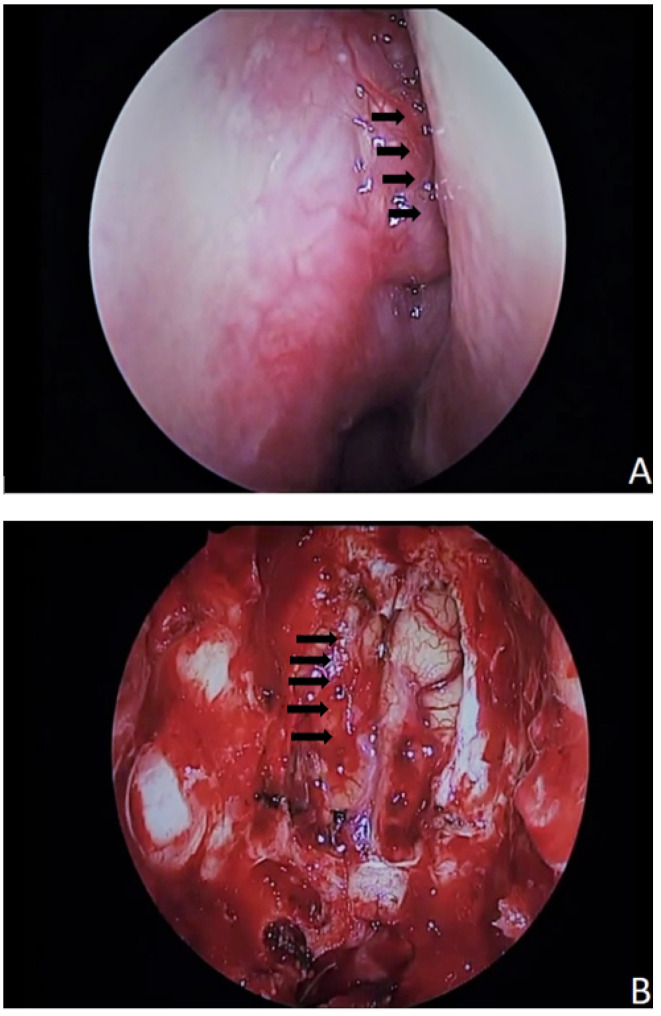
Intraoperative photos of the same case with olfactory neuroblastoma. (**A**) Endoscopic picture of the tumorous lesion (black arrows) in the right nasal cavity. (**B**) Surgical situs after sole endoscopic resection of the olfactory neuroblastoma, bilateral resection of the olfactory bulbs, and the remaining intracranial portion of the tumor (black arrows).

A thorough search of the relevant literature reveals that prospective data remain elusive because of the extremely low incidence of this entity [27]. The increased incidence in the literature of recent years mirrors the improved diagnostic capacity and underlines the necessity for well-designed treatment algorithms [28]. The first issue to deal with is the form of management of the primary tumor. A review of the literature, as well as an investigation of our data, revealed a shift of paradigm from the “gold standard” of craniofacial resection in recent decades of the last century to the continuously increasing performance of endoscopic resection (as a sole or adjunct approach), with some centers even employing endoscopic techniques for the resection of selected tumors with intracranial extension (Figure 5 and Figure 6) [29,30]. In any case, a multimodal approach (surgery with adjuvant irradiation) is thought to be the “gold standard” for high-risk cases (e.g., R1 situation, advanced Kadish stages, aggressive Hyams subtypes) in treatment protocols intended to cure. While the role of chemotherapy is not well defined [30], it could, however, play a role in neoadjuvant settings for locally advanced tumorous lesions [19,31] and cases with primary distant metastases or distant recurrences [32]. Several literature reports point out the possibility of surgery as the sole form of treatment in very carefully selected cases [19]. Examining the subgroup of our study patients with surgery as the sole form of management (6/42, 14.3%), we saw that three patients (50%) died of the disease (among them, one with Kadish stage C and one with Hyams IV). Meerwein et al. saw a potential reduction in therapeutic invasiveness (surgery as a single-modality treatment) in cases with the following profile: limited local tumor extension (Kadish stage A–B), absence of brain involvement, Hyams grade up to III, and microscopically clear surgical margins (based on a definitive histopathological workup) [19]. An investigation of our data revealed a shift of paradigm in the treatment of these tumors following the development and expansion of the spectrum of endoscopic surgery for this indication three decades ago. It seems that surgery alone could only be an equal alternative to multimodal treatment in carefully selected patients with a lack of risk factors and after a thorough discussion of each case with an interdisciplinary tumor board.

A reasonable treatment algorithm should be based on thorough knowledge of the biologic (metastatic) behavior of the disease. In this context, Koch et al. detected local recurrences in 23% of their study cases [3]. Similarly, Constantinidis et al. found local recurrences in 19.2% of their patient sample [33]. In our study, local recurrence was detected in 8/53 of the study cases (15.1%). The vast majority of these cases (7/8) had a Kadish stage higher than B and a Hyams grade higher than III. Three cases were managed (in the first years of the study) by means of neoadjuvant radio(chemo)therapy and two with adjuvant irradiation after primary surgery. Reasonably, local recurrence reflects a highly aggressive form of the disease, with the majority of the cases (4/8) dying within a short period of time (8–26 months) after the initial diagnosis of the tumor.

According to the relevant literature, 5–8% of patients with ON show metastatic involvement of the neck at the time of initial presentation [3,34,35]. In our study sample, regional involvement at the time of the first diagnosis was almost 10%. This percentage scale is certainly below the “20% law” for elective treatment of the regional lymphogenous network that was described by Weiss et al. [36], pointing to the fact that a possible “wait-and-scan” policy without management of the lymphatic stations might be sufficient in the majority of cases. However, this percentage does not justify complacency and makes a thorough scan of the neck within the initial diagnostic workout inevitable. Admittedly, the involvement of the neck gains much more importance in the form of regional recurrence at a later stage in the course of disease, with an incidence as high as 25% [34]. The incidence of this parameter in our long-term analysis (7/53, 13.2%) was almost the same as that seen in a systematic review by Naples et al. [12]. A careful investigation of this subgroup of patients revealed the consistent presence of “high-risk” tumor characteristics, namely higher Kadish stages (three cases with Kadish stage B and three with C) and advanced Hyams grades (higher than II) in all cases. Six out of these seven cases had received irradiation of the primary lesion (neoadjuvant in one case with Kadish stage C and Hyams grade III, adjuvant in the remaining five cases). In two cases, the regional failure was combined with local relapse, and the disease showed massive progression with subsequent distant metastases and the patient’s death after 8 and 21 months after initial diagnosis. In the remaining five cases with solitary regional recurrences, the mean time to regional recurrence was 83 months (68–96 months). Our data showed that management of the invaded lymph nodes by means of neck dissection with adjuvant radio (chemo) therapy could achieve an acceptable long-term oncologic result (Table 1).

**Figure 7:**
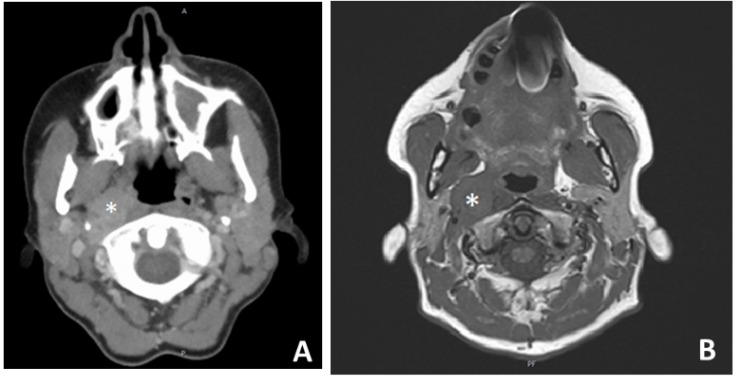
Axial contrast-enhanced computed tomography (**A**) and T1-weighted MRI (**B**) of a patient with regional recurrence in the right parapharyngeal space (white asterisk) 68 months after initial craniofacial resection and adjuvant irradiation in olfactory neuroblastoma Kadish–Morita stage B/Hyams grade III.

The ongoing controversy in the management of a clinically negative neck in ON [7] is reflected, among others, in a popular radiation oncology textbook that states, on the one hand, that “the available data do not justify routine elective nodal treatment”, but recommends in another Section, 123 pages later, that “with advanced-stage disease, cervical lymph nodes should be initially managed by irradiation, radical neck dissection, or a combination of both” [37]. Elective neck irradiation was not administered routinely to a cN0 neck in either of the departments involved in the present study. However, as patients with regional recurrences tend to have higher mortality [12] (worse survival outcomes) and given that “prevention is the best treatment”, our aforementioned observations sustain the reasonability of elective neck management (e.g., irradiation) in specific cases in which risk-stratified adjuvant irradiation of the primary tumor site is indicated [38]. In other words, if a local finding has such aggressive features that it has to be irradiated, then a cN0 neck will probably also have to be irradiated, as both the primary tumor and the regional lymphatic network belong to the same case of a “high-risk” profile! Eighteen years ago, Constantinidis et al. pointed to the frequent development of regional recurrences, sometimes long after initial therapy, independent of any type of aggressive therapy [33]. Another interesting observation was that in 2/7 cases with regional recurrence, the positive lymph node was localized in the retro- and parapharyngeal space (Figure 7). The already described rather rare tendency of the olfactory neuroepithelium tumor cells to metastasize in the lymphatic network of the retro- and parapharyngeal space [39,40] necessitates radiologic vigilance both in the initial staging and in the follow-up. In a relevant literature report [39], as well as in one of our cases, the pathologic changes in the lymph nodes, in retrospect, were already present on the first images of the axial datasets and were initially overlooked on routine MRI evaluation of the neck. Potential involvement of the retro- or parapharyngeal lymph nodes (Figure 7) has both a clinical relevance as well as a radiologic implication: First of all, it seems reasonable that primary management of the cN0 neck, if indicated, has to take the form of irradiation, as an elective neck dissection alone cannot easily address the (in case of recurrence frequently usually involved) retro- and parapharyngeal space without a significant increase in surgical morbidity [41]. Secondly, it gives computer tomography, MRI, or FDG-PET/CT the lead over ultrasound in the imaging of the neck. Considering the fact that an N+ situation changes the stage to Kadish–Morita D [26], worsens the prognosis [12,35], and definitively justifies adjuvant irradiation in the initial phase [42], it cannot be emphasized enough that a thorough initial scan of the patient is of major importance.

## 5. Conclusions

Firstly, the survival analysis of our study showed superior cumulative DSS for limited lesions (no significance) and low-grade tumors (highly significant). A review of the relevant literature reveals a more consistent position concerning the prognostic importance of the Kadish staging system [19] but more variability concerning the prognostic impact of the histologic grading [16]. Secondly, a statistically better survival was detected in the group of patients being treated in the latter study period (with cases of advanced stage as well as a higher grade being homogeneously distributed in both study groups), pointing to the positive impact of the increasing experience in the oncologic outcome of our cases. Thirdly, the non-negligible incidence of regional recurrences, partly in unusual localizations (e.g., retro- and parapharyngeal space), leads us to consider the need for identifying the “recurrence-friendly” cases and for primary elective irradiation of the neck in cases with high-risk features. Interestingly, the present long-term study confirms the reliability of the results of an analysis of one of the involved departments 18 years ago [33]. Last but not least, individualization of this indication with consideration of other factors (e.g., age) is needed.

## Figures and Tables

**Table 1:** Patient, tumor, and treatment characteristics of all patients of our study sample (ESS: endoscopic sinus surgery, TFA: transfacial approach (lateral rhinotomy), BFC: bifrontal craniotomy, aRT: adjuvant irradiation, RCT: Radiochemotherapy, AND: alive and free of disease, AWD: alive with disease, DOD: dead because of disease, DAD: dead for non-disease-relevant reason).

ID	Gender	Age (y)	Stage (Kadish)	Stage (Kadish–Morita)	Histologic Grading (Hyams)	N Status	Treatment	Recurrence (After … Months)	Outcome (Follow-Up in Months)
**1**	Female	10	B	B	IIII	N0	Neoadjuvant RCT + BFC	Local	AND (144)
**2**	Male	56	B	B	III	N0	Neoadjuvant RT + BFC	Local	DAD (259)
**3**	Female	22	C	D	IV	N3	Neoadjuvant RCT + BFC	No	DOD (4)
**4**	Female	38	C	C	III	N0	Neoadjuvant RT + TFA	Locoregional	DOD (21)
**5**	Male	59	A	A	II	N0	ESS	No	AND (199)
**6**	Male	54	B	B	I	N0	ESS	No	AND (156)
**7**	Female	63	B	B	II	N0	ESS	No	AND (120)
**8**	Female	77	B	B	IV	N0	TFA	Local	DOD (14)
**9**	Female	46	C	C	III	N0	BFC	No	AND (122)
**10**	Female	50	B	B	III	N0	BFC	Locoregional	DOD (8)
**11**	Male	50	C	C	III	N0	BFC	No	DOD (4)
**12**	Male	28	B	B	II	N0	ESS + aRT	No	AND (214)
**13**	Female	67	B	B	II	N0	ESS + aRT	No	DAD (131)
**14**	Male	16	B	B	III	N0	ESS + aRT	No	AND (141)
**15**	Male	52	B	D	III	N1	ESS + aRT	No	AND (124)
**16**	Male	36	C	C	III	N0	ESS + aRT	No	AND (208)
**17**	Male	48	C	C	III	N0	ESS + aRT	Regional	AND (189)
**18**	Female	55	C	C	III	N0	ESS + aRT	No	AND (154)
**19**	Female	41	C	C	III	N0	ESS + aRT	No	AND (115)
**20**	Male	71	B	B	III	N0	ESS + aRT	No	DAD (132)
**21**	Male	27	B	B	II	N0	ESS + aRT	Regional	AND (180)
**22**	Female	56	B	B	III	N0	ESS + aRT	No	AND (120)
**23**	Female	64	C	D	II	N1	ESS + BFC+ aRT	No	AND (205)
**24**	Female	62	C	C	I	N0	ESS + BFC+ aRT	No	AND (75)
**25**	Male	80	B	B	I	N0	ESS + BFC + aRT	No	DAD (143)
**26**	Male	57	A	A	II	N0	ESS + BFC + aRT	No	AND (264)
**27**	Female	53	B	B	II	N0	ESS + BFC + aRT	No	AND (300)
**28**	Male	32	C	C	III	N0	ESS + BFC + aRT	Local	DOD (26)
**29**	Male	15	B	B	II	N0	TFA + aRT	No	AND (288)
**30**	Male	51	B	B	I	N0	BFC + aRT	Local	AND (156)
**31**	Male	48	C	C	I	N0	BFC + aRT	No	AND (264)
**32**	Female	57	C	C	I	N0	BFC + aRT	No	AND (252)
**33**	Male	62	C	C	I	N0	BFC + aRT	No	DAD (117)
**34**	Female	38	B	B	II	N0	BFC + aRT	Regional	AND (192)
**35**	Female	31	B	B	II	N0	BFC + aRT	No	AND (180)
**36**	Female	84	B	B	II	N0	BFC + aRT	No	DAD (16)
**37**	Male	36	C	C	III	N0	BFC + aRT	No	AND (336)
**38**	Female	34	C	D	III	N3	BFC + aRT	Regional	AND (180)
**39**	Female	45	C	C	III	N0	BFC + aRT	No	AND (123)
**40**	Female	43	C	C	III	N0	BFC + aRT	No	AND (126)
**41**	Male	68	C	C	III	N0	BFC + aRT	Distant recurrence	AND (144)
**42**	Female	69	C	C	IV	N0	BFC + aRT	No	AND (259)
**43**	Female	17	B	B	III	N0	ESS + aRT	No	AND (96)
**44**	Male	51	C	C	III	N0	ESS + aRT	No	AND (84)
**45**	Male	24	C	C	IV	N0	ESS + BFC + aRT	No	AND (70)
**46**	Female	44	B	B	III	N0	ESS +aRT	No	AND (64)
**47**	Male	62	A	A	II	N0	ESS + aRT	No	AND (58)
**48**	Male	55	C	C	III	N2	Neoadjuvant RCT + ESS	No	AND (47)
**49**	Female	65	C	C	II	N0	ESS + BFC + aRT	No	AND (61)
**50**	Male	53	B	B	II	N0	ESS + aRT	No	AND (84)
**51**	Female	67	B	B	III	N0	ESS + aRT	No	AND (93)
**52**	Female	43	C	C	III	N0	ESS + BFC + aRT	Local, distant	AWD (104)
**53**	Male	47	B	B	III	N0	ESS + aRT	Regional	AND (124)

**Table 2:** Detailed information of all study patients and treatment characteristics.

**Gender (*n*, %)**	
Male	26 (49.1)
Female	27 (50.9)
**Kadish** [8] **stage (*n*, %)**	
A	3 (5.7)
B	25 (47.2)
C	25 (47.2)
**Kadish–Morita** [26] **grading (*n*, %)**	
A	3 (5.7)
B	24 (45.3)
C	22 (41.5)
D	4 (7.5)
**Hyams** [13] **(*n*, %)**	
I	7 (13.2)
II	1 (28.3)
III	26 (49.1)
IV	5 (9.4)
**Nodal stage (*n*, %)**	
N0	48 (90.6)
N+	5 (9.4)
**Therapeutic approach (*n*, %)**	
Surgery only	7 (13.2)
Surgery + adjuvant irradiation	41 (77.3)
Neoadjuvant R(C)T + surgery	5 (9.4)
**Surgical approach (*n*, %)**	
Endoscopic only	22 (41.5)
Endoscopic + open (craniofacial)	22 (41.5)
Open (craniofacial)	9 (17)

**Table 3:** Disease-specific survival estimates for all study patients stratified by Kadish–Morita stage and Hyams grading.

KERRYPNX	5-Year Disease-Specific Survival Estimates	*p*-Value
**Kadish–Morita** [26] **stage**		
A–B	92.6%	0.377
C–D	84.6%	
**Hyams** [13] **grading**		
I–II	100%	
III–IV	80.6%	**0.32**

## Data Availability

The data presented in this study are available on request from the corresponding author.
